# Hydralazine-induced anti-neutrophil cytoplasmic antibody-positive renal vasculitis presenting with a vasculitic syndrome, acute nephritis and a puzzling skin rash: a case report

**DOI:** 10.1186/1752-1947-7-20

**Published:** 2013-01-14

**Authors:** Justin Keasberry, Jeremy Frazier, Nicole M Isbel, Carolyn L Van Eps, Kimberley Oliver, David W Mudge

**Affiliations:** 1 Department of Nephrology, University of Queensland at Princess Alexandra Hospital, Brisbane, Australia; 2 Department of Nephrology, Logan Hospital, Brisbane, Australia; 3 Department of Anatomical Pathology, Princess Alexandra Hospital, Brisbane, Australia

**Keywords:** ANCA, Drug-induced vasculitis, Hydralazine, Vasculitis

## Abstract

**Introduction:**

Anti-neutrophil cytoplasmic antibody-associated vasculitis has been associated with many drugs and it is a relatively rare side effect of the antihypertensive drug hydralazine. The diagnosis and management of patients who have anti-neutrophil cytoplasmic antibody-associated vasculitis may be challenging because of its relative infrequency, variability of clinical expression and changing nomenclature. The spectrum of anti-neutrophil cytoplasmic antibody-associated vasculitis is wide and can be fatal. This case documents a 62-year-old woman who presented with hydralazine-induced anti-neutrophil cytoplasmic antibody-positive renal vasculitis with a puzzling cutaneous rash.

**Case presentation:**

We report a rare case of hydralazine-induced anti-neutrophil cytoplasmic antibody-associated vasculitis in a 62-year-old Caucasian woman who presented with a vasculitic syndrome with a sore throat, mouth ulcers and otalgia after several months of constitutional symptoms. She then proceeded to develop a rash over her right lower limb. Clinically, the rash had features to suggest Sweet’s syndrome, but also had some appearances consistent with embolic phenomena and did not have the appearance of palpable purpure usually associated with cutaneous vasculitis. Differential diagnoses were hydralazine-associated Sweet’s syndrome, streptococcal-induced cutaneous eruption or an unrelated contact dermatitis. A midstream urine sample detected glomerular blood cells in the setting of anti-neutrophil cytoplasmic antibody-positive renal vasculitis and *Streptococcus pyogenes* bacteremia. A renal biopsy revealed a pauci-immune, focally necrotizing glomerulonephritis with small crescents. Her skin biopsy revealed a heavy neutrophil infiltrate involving the full thickness of the dermis with no evidence of a leucocytoclastic vasculitis, but was non-specific. She was initially commenced on intravenous lincomycin for her bloodstream infection and subsequently commenced on immunosuppression after cessation of hydralazine. The patient was subsequently discharged from hospital after a rapid clinical improvement.

**Conclusion:**

Hydralazine-induced anti-neutrophil cytoplasmic antibody-positive renal vasculitis is a rare adverse effect and can present with a severe vasculitic syndrome with multiple organ involvement. Features of this association include the presence of high titres of anti-myeloperoxidase-anti-neutrophil cytoplasmic antibody with multi-antigenicity, positive anti-histone antibodies and the lack of immunoglobulin and complement deposition histopathogically. A rash that is characteristic of Sweet’s syndrome has also been described as an association. Prompt cessation of hydralazine may be sufficient to reverse disease activity but immunosuppression may be needed for definite treatment.

## Introduction

Hydralazine is a vasodilator that is often used as an adjunctive agent in the treatment of hypertension. Anti-neutrophil cytoplasmic antibody-associated vasculitis has been associated with many drugs such as allopurinol, sulfasalazine and propylthiouracil and is a relatively rare side effect of hydralazine. The etiology of ANCA-associated vasculitis (AAV) is not always clear and this association is less well recognized compared to drug-induced lupus which is well documented in the literature [1]. The diagnosis and management of patients may be challenging because of its relative infrequency, variability of clinical expression and changing nomenclature. The spectrum of AAV can range from cutaneous rashes and petechiae or single organ involvement to fatal multi-organ involvement with deaths commonly from massive pulmonary hemorrhage. A latency of several years can occur before the development of vasculitis with a variable delay in the full clinical manifestations [2] therefore posing a challenge for clinicians to achieve clear diagnoses and treatment strategies. This case documents a 65-year-old woman who presented with a diagnostic dilemma after developing a lower limb rash and a sore throat with *Streptococcus pyogenes* bacteremia and highlights the need for early recognition to enable timely cessation of the offending drug or drugs and commencement of appropriate therapy.

## Case presentation

**Figure 1:**
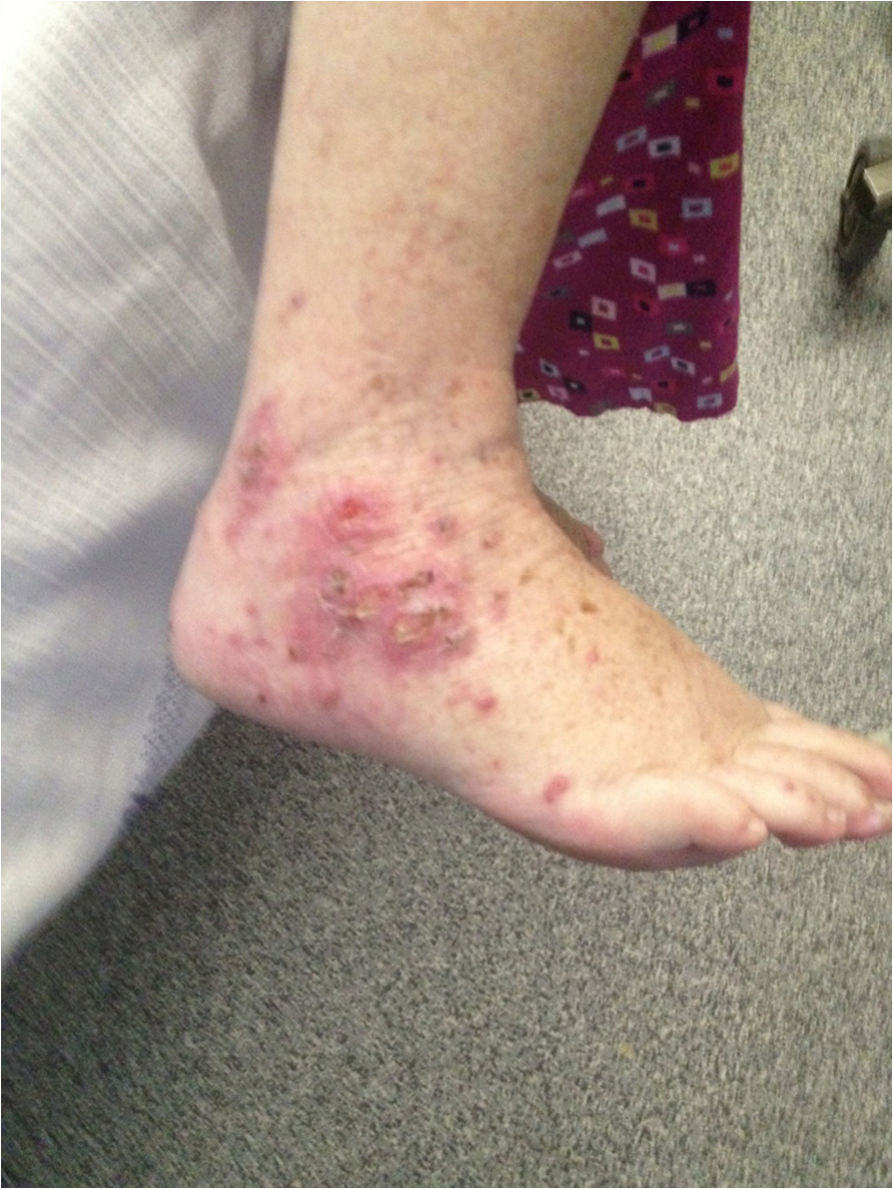
Photograph of the right ankle and foot showing an erythematous papular rash with ulcerations on presentation.

**Figure 2:**
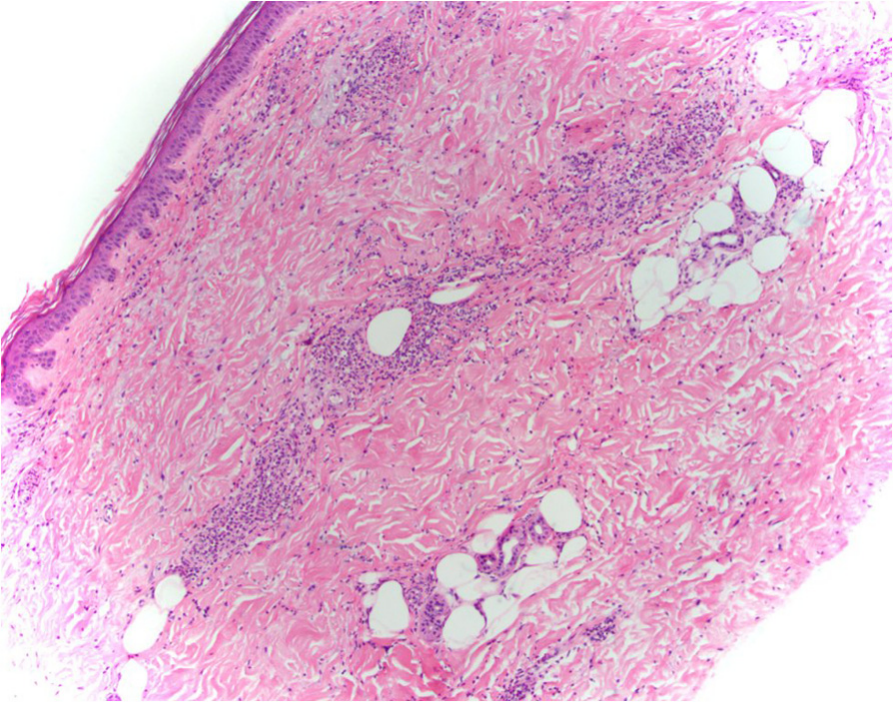
Photomicrograph of the skin biopsy with a hematoxylin and eosin stain ×100 showing a heavy neutrophil infiltrate involving the full thickness of the dermis with no evidence of a leucocytoclastic vasculitis.

**Figure 3:**
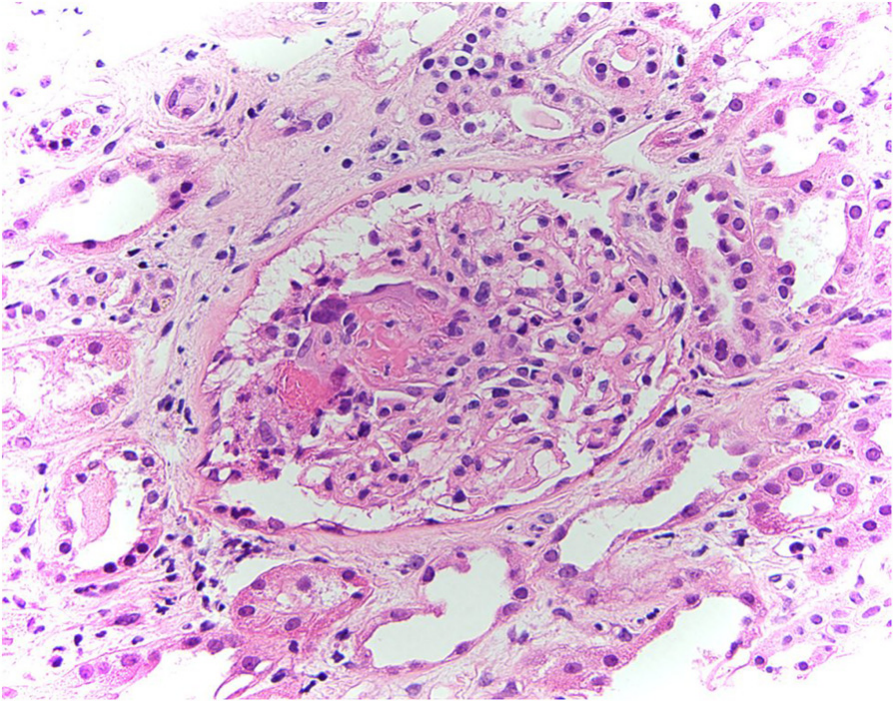
**Photomicrograph of the renal biopsy with a hematoxylin and eosin stain ×400 magnification showing a pauci-immune, focal necrotizing glomerulonephritis with one small crescent consistent with anti-neutrophil cytoplasmic antibody-associated vasculitis.** Red cell casts are present. There is an interstitial infiltrate of lymphocytes, macrophages and eosinophils which involves some tubules. Immunofluorescence staining revealed mild mesangial deposition of immunoglobulin M and traces of mesangial C3.

A 62-year-old Caucasian woman of Anglo-Saxon background with resistant hypertension developed a sore throat, mouth ulcers and otalgia after several months of constitutional symptoms consisting of lethargy, night sweats and significant weight loss. She then proceeded to develop a rash over her right lower limb. Her past medical history consisted of Hashimoto’s thyroiditis, rheumatic heart disease, hyperlipidemia and recurrent deep venous thromboses due to the factor V Leiden mutation for which she was on lifelong warfarin. Other medications taken regularly consisted of: hydralazine 100mg in the morning, 50mg at night which she had taken for three years; perindopril 10mg daily; metoprolol 50mg twice daily; clonidine 50mg twice daily; thyroxine 50mcg and 25mcg on alternate days; and rosuvastatin 5mg at night. An examination revealed hypertension (blood pressure 160/70mmHg), a pansystolic murmur, an aphthous ulcer at the base of her tongue and a rash over her right ankle and foot (Figure 1). Baseline blood tests revealed a leucocytosis of 19.2×10^9^/L with a neutrophilia of 16.85×10^9^/L and raised erythrocyte sedimentation rate of 123mm/hour. Blood cultures taken on admission yielded *S. pyogenes* and she was immediately treated with intravenous lincomycin because of a previous penicillin allergy. A transesophageal echo was carried out which excluded infectious endocarditis. Blood tests revealed impaired kidney function, a serum creatinine concentration of 102μmol/L, and an estimated glomerular filtration rate (eGFR) of 48mL/min/1.73m^2^ which is at her baseline renal function. Her urine revealed microscopic hematuria with 360×106/L red blood cells without any urinary tract infection and she only had mild proteinuria of 460mg/L, and a protein:creatinine ratio of 48g/mol. Further investigations revealed an anti-nuclear antibody >2560, double-stranded deoxyribonucleic acid (DNA) antibody elevated at 14 (<7), perinuclear (P)-ANCA >2560 and anti-myeloperoxidase (MPO) antibody >100μ/mL with positive anti-histone antibodies. Because of glomerular blood cells in a repeat midstream urine sample taken on day 17 after presentation, acute kidney injury with a serum creatinine of now 195μmol/L, eGFR 23mL/min/1.73m^2^, worsening proteinuria (810mg/L), normal serum complement levels and high titres of vasculitic markers, a drug-associated ANCA vasculitis was suspected. However, due to the temporal relation of two weeks between a streptococcal bloodstream infection and the development of an acute nephritic syndrome, post-streptococcal glomerulonephritis was also considered. The associated skin rash (Figures 1 and 2) did not have a clear role in her presentation but was initially thought to be early Sweet’s syndrome associated with a drug-associated vasculitis. Hydralazine was withdrawn and the patient underwent a transjugular renal biopsy to confirm the diagnosis (Figure 3). The patient was commenced on treatment consisting of three intravenous pulses of methylprednisolone followed by high dose prednisolone and mycophenolate mofetil 1g twice daily. Her urine immediately became clear and her skin rash resolved quickly when the mycophenolate mofetil was started and her renal function improved, suggesting that the cause of the rash was immunological in etiology. Serum creatinine concentration on discharge (day 35) was 144μmol/L. The patient attended an out-patient follow-up clinic a fortnight and then a month later to commence prednisone tapering as per the European Vasculitis Study Group protocol and to continue her mycophenolate mofetil for six months. A repeat serum creatinine concentration was 96μmol/L and anti-MPO was 16u/mL.

## Discussion

Hydralazine-induced ANCA vasculitis is rare and not the first diagnosis that came to mind with our patient who displayed a wide spectrum of disease of drug-induced AAV with severe renal and possible cutaneous manifestations requiring immunosuppression. The presence of high titres of P-ANCA and anti-MPO with multi-antigenicity, the positive anti-histone antibodies and the lack of immunoglobulin and complement deposition histopathogically are features that have been described with drug-induced ANCA vasculitis [2,3] rather than with drug-induced lupus or with primary vasculitis. Therefore we strongly suspect this to be an adverse effect of hydralazine. The histology for the cutaneous rash was not classical for Sweet’s syndrome which would involve a denser neutrophilic infiltration in the dermis; however, the cutaneous rash did have some similarities with this syndrome: a neutrophilic infiltration on histology, a neutrophilia on presentation, fever and a tender papular skin rash. This rash was also in the setting of a *S. pyogenes* bacteremia which could have resulted in the skin rash. The differential diagnoses for the cutaneous rash were hydralazine associated with early Sweet’s syndrome as described with carbamazepine in the literature [4], cutaneous vasculitis [5], erythroderma, streptococcal-induced cutaneous eruption from hematogenous spread, or an unrelated cause such as a contact dermatitis or early cellulitis.

Risk factors that have been identified predisposing to hydralazine-related AAV include a cumulative dose of more than 100g, female sex, and history of thyroid disease [6] as seen in our patient. Other risk factors include the human leukocyte antigen (HLA)-DR4 genotype, slow hepatic acetylation and the null gene for C4 [7]. The mechanism or mechanisms for hydralazine-induced AAV is not fully understood but might be multifactorial. Hypotheses of immune system activation by drug metabolites with autoimmunity towards neutrophil proteins (including elastase and lactoferrin) and upregulation of ANCA antigens have been suggested [5].

A review of the literature revealed 68 patients with hydralazine-induced vasculitis [1,8,9] and five cases with ANCA-positive hydralazine-associated immune complex-mediated renal vasculitis with cutaneous involvement. However, there were no cases of ANCA-positive hydralazine-associated renal vasculitis with pauci-immune glomerulonephritis and a vasculitic cutaneous rash or Sweet’s syndrome (see Table 1). The hydralazine dosage ranged from 50 to 300mg per day, and the treatment duration varied from 0.73 to 120 months. Almost all the patients from the literature were positive for MPO antibodies and all of the patients with anti-histone antibodies checked had positive results. Our patient did not have clear cutaneous involvement with her presentation and the aforementioned renal–cutaneous association syndrome is yet to be published in the literature to date.

**Table 1:** Hydralazine-associated anti-neutrophil cytoplasmic antibody-positive pauci-immune glomerulonephritis

	**Short and Lockwood (1995) [**[10]**]**	**Choi *et al*. (2000) [**[1]**]**	**Yokogawa and Vivino (2009) [**[9]**]**
Number of patients	10	10	68
Age range (years)	55–76	45–81	60–67
Females (%), female/total patients	70 (7/10)	70 (7/10)	59 (40/68)
Hydralazine dose range (mg/day)	50–150	75–200	50–300
Duration of drug exposure (months)	36–156	12–63	0.73 to >120
Patients with renal involvement	10	9	55
Patients with cutaneous involvement	4	3	25
Patients with pulmonary involvement	2	5	13
Confirmed pauci-immune GN	7	5	>25 *
ANCA-positive	10	10	45
MPO-positive	10	10	45
Anti-histone-positive (patients)	NR	NR	(12/12)

Abbreviations: ANCA, anti-neutrophil cytoplasmic antibodies; GN, glomerulonephritis; MPO, myeloperoxidase antibodies; NR, not reported.

* Authors estimated number from data available in the literature.

## Conclusion

Hydralazine-induced ANCA-positive renal vasculitis is a rare adverse effect and can present with a severe vasculitic syndrome with multiple organ involvement. Early diagnosis and recognition of the clinical patterns of disease will be essential for prompt treatment. Timely cessation of hydralazine may be sufficient to reverse disease activity to prevent progression of disease towards end-stage renal failure [11]. However, a short course of immunosuppressive therapy and monitoring of serum ANCA may be all that is required without the need for long-term maintenance. Caution should be displayed when using this drug and it may be prudent to refrain from prescribing it to patients with a past history of autoimmune disease and to regularly check for urinary glomerular blood cells.

## Consent

Written informed consent was obtained from the patient for publication of this case report and accompanying images. A copy of the written consent is available for review by the Editor-in-Chief of this journal.

## Abbreviations

AAV: ANCA-associated vasculitis; ANCA: Anti-neutrophil cytoplasmic antibody; eGFR: Estimated glomerular filtration rate; MPO: Myeloperoxidase; P-ANCA: Perinuclear-ANCA.

## Competing interests

The authors declare that they have no competing interests.

## Authors’ contributions

JK, JF, NI, CvE and DM cared for the patient during her hospital stay. NI and CvE reviewed the patient’s history, signs, laboratory data and investigations and made the diagnosis of hydralazine-associated ANCA-positive renal vasculitis. JK, JF and DM wrote the manuscript and performed a literature search. KO examined the skin and renal histology and provided a report on the photomicrographs. All authors have read, edited and approved the final manuscript.
